# Genome Sequence of *Acidovorax citrulli* Group 1 Strain pslb65 Causing Bacterial Fruit Blotch of Melons

**DOI:** 10.1128/genomeA.00327-15

**Published:** 2015-04-23

**Authors:** Tielin Wang, Baixin Sun, Yuwen Yang, Tingchang Zhao

**Affiliations:** State Key Laboratory for Biology of Plant Diseases and Insect Pests, Institute of Plant Protection, Chinese Academy of Agricultural Sciences, Beijing, China

## Abstract

*Acidovorax citrulli* is typed into two groups, mainly based on the host. We determined the draft genome of *A. citrulli* group 1 strain pslb65. The strain was isolated from melon collected from Xinjiang province, China. The *A. citrulli* pslb65 genome contains 4,903,443 bp and has a G+C content of 68.8 mol%.

## GENOME ANNOUNCEMENT

The worldwide seedborne disease bacterial fruit blotch (BFB) is caused by the Gram-negative bacterium *Acidovorax citrulli* (1, 2). Previous reports showed that *A. citrulli* was typed into two groups, mainly based on the host: group 1 includes strains mainly from melon, and group 2 includes strains mainly from watermelon (3, 4). Earlier research showed that there were many differences of copper resistance between strains in the two groups (5). Bahar et al. also showed that pili genes were different between the two groups (6). In 2007, the draft genome sequence of *A. citrulli* AAC00-1 (NCBI GenBank accession no. NC_008752), which belongs to group 2, was reported. For the further studies on the pathogenic mechanism and genetic diversity of the pathogen that causes BFB, we report here the draft genome of the group 1 strain *A. citrulli* pslb65, which was isolated in August of 2009 from a melon seedling displaying leaf scorch symptoms in Xinjiang province, China.

The genome of this strain was sequenced with MPS (massively parallel sequencing) Illumina technology. Two DNA libraries were constructed: a paired-end library with an insert size of 300 to 400 bp and a mate-pair library with an insert size of 5 kb. Using Velvet version 1.2.07, we assembled the genome into 23 scaffolds. The largest scaffold was 720,064 bases. Among the large scaffolds, the *N*_50_ scaffold size was 573,229 bases. The scaffolds have an average length of 204,310 bases. Gene prediction on the *A. citrulli* strain AAC00-1 genome assembly was performed with GeneMarkS (http://topaz.gatech.edu) (7). A whole-genome BLAST search (E-value ≤1E-5, minimal alignment length percentage ≥40%) was performed against five databases: TREMBL (computer-annotated supplement to Swiss-Prot) (8), NR (Non-Redundant Protein) (9), Swiss-Prot (10), COG (Clusters of Orthologous Groups) (11), and GO (Gene Ontology) (12).

The genome contains a single circular chromosome of 4,903,443 bp with a GC content of 68.8%. The coding genes totaled 4,344,750 bp; 4,532 protein-coding genes are identified in the genome with an average length of 958 bp. There are 3 rRNAs and 51 tRNAs.

### Nucleotide sequence accession numbers. 

This whole-genome shotgun project has been deposited at DDBJ/EMBL/GenBank under the accession number JYHM00000000. The version described in this paper is version JYHM01000000.
